# Implementing the MOVE! Weight-Management Program in the Veterans Health Administration, 2007-2010: A Qualitative Study

**Published:** 2011-12-15

**Authors:** Bryan J. Weiner, Lindsey Haynes-Maslow, Marci K. Campbell, Leila C. Kahwati, Linda S. Kinsinger

**Affiliations:** Department of Health Policy and Management, Gillings School of Global Public Health, University of North Carolina at Chapel Hill; University of North Carolina at Chapel Hill, Chapel Hill, North Carolina; University of North Carolina at Chapel Hill, Chapel Hill, North Carolina; Veterans Health Administration, Durham, North Carolina; Veterans Health Administration, Durham, North Carolina

## Abstract

**Introduction:**

One-third of US veterans receiving care at Veterans Health Administration (VHA) medical facilities are obese and, therefore, at higher risk for developing multiple chronic diseases. To address this problem, the VHA designed and nationally disseminated an evidence-based weight-management program (MOVE!). The objective of this study was to examine the organizational factors that aided or inhibited the implementation of MOVE! in 10 VHA medical facilities.

**Methods:**

Using a multiple, holistic case study design, we conducted 68 interviews with medical center program coordinators, physicians formally appointed as program champions, managers directly responsible for overseeing the program, clinicians from the program's multidisciplinary team, and primary care physicians identified by program coordinators as local opinion leaders. Qualitative data analysis involved coding, memorandum writing, and construction of data displays.

**Results:**

Organizational readiness for change and having an innovation champion were most consistently the 2 factors associated with MOVE! implementation. Other organizational factors, such as management support and resource availability, were barriers to implementation or exerted mixed effects on implementation. Barriers did not prevent facilities from implementing MOVE! However, they were obstacles that had to be overcome, worked around, or accepted as limits on the program's scope or scale.

**Conclusion:**

Policy-directed implementation of clinical weight-management programs in health care facilities is challenging, especially when no new resources are available. Instituting powerful, mutually reinforcing organizational policies and practices may be necessary for consistent, high-quality implementation.

## Introduction

In 2006, the Veterans Health Administration (VHA) issued a policy directing implementation of an evidence-based weight-management program to help reduce obesity rates among veterans receiving care from VHA (1). Created by VHA's National Center for Health Promotion and Disease Prevention (NCP) on the basis of guidelines from the National Institutes of Health (2,3) and other literature, the MOVE! weight-management program uses a population-based clinical approach to disease in which all patients seen in VHA medical facilities are systematically screened for obesity and offered evidence-based tiered treatment options tailored to their needs and preferences. In order of increasing intensity, treatment options include self-management support, individual counseling or group sessions, clinically supervised weight-management medications, and, in some facilities, brief residential treatment or bariatric surgery. Delivered by a multidisciplinary team encompassing primary care, dietetics, behavioral health, and physical activity, MOVE! is a comprehensive approach to weight loss and maintenance that promotes behavior change, healthy nutrition, physical activity, and psychological well-being. MOVE! addresses an urgent need: 35% of VHA primary care enrollees — representing 90% of all of VHA patients — are estimated to be obese (4,5) and, therefore, at higher risk for chronic diseases such as hypertension, cardiovascular disease, stroke, and osteoarthritis (6).

NCP took several steps in designing and disseminating MOVE! to ensure rapid adoption and implementation (7). First, it developed an easy-to-use toolkit that contained patient handouts, promotional brochures, clinical references, curriculum modules, online staff training, implementation checklists, administrative manuals, and marketing materials. Second, it tested the program for feasibility in 17 VHA medical facilities and revised program content and materials on the basis of staff and patient feedback. Third, NCP secured endorsements for the program from influential internal stakeholders, culminating in the issuance of a VHA policy in March 2006 requiring all facilities to implement MOVE! or an equivalent multidisciplinary weight-management program. Fourth, NCP held 2 national training conferences and biweekly teleconferences with program coordinators in the 21 regional VHA networks. Finally, VHA policy required facilities to complete an annual report on their weight-management services and prepare to be held accountable for their obesity screening rates as part of VHA's performance measurement system.

By 2009, nearly all (98.7%) of the 155 medical centers in VHA reported having MOVE! programs in place (7). A VHA evaluation conducted in 2010 showed that, overall, the program has had a modestly positive effect on weight change at 6 months (8). However, facilities varied in the speed with which they implemented the program and the level of program activity they achieved 12 to 36 months after the issuance of the policy. Given the national scope of the program's dissemination within a single health care system, the MOVE! program offers a unique opportunity to examine the local organizational factors that aided or hindered program implementation among multiple facilities. The objective of this study was to examine the organizational facilitators and barriers of MOVE! implementation in 10 VHA medical facilities.

## Methods

### Conceptual framework

We used an organizational model of innovation implementation to guide the study (9-11). The model posits that the effective implementation of an innovation (ie, consistent, high-quality delivery of MOVE!) is a function of the organization's readiness for change; level of management support for the innovation; amount of resources available for implementation; presence of an innovation champion; extent to which the innovation fits local task demands, such as work processes and patient preferences ("innovation-task fit"); and extent to which intended implementers of the innovation, such as physicians, nurses, and allied health professionals, perceive that innovation implementation fosters the fulfillment of their values ("innovation-values fit").

### Study design and sample

We used a multiple, holistic case study design; the VHA medical facility was the unit of analysis (12). Case study methods are well suited for studying implementation processes, which tend to be fluid, nonlinear, and context-sensitive (13-15). We invited 126 VHA facilities with at least 30 active MOVE! participants in 2006 to participate in our study. Of the 14 facilities that accepted our invitation, we purposefully selected 10 to reflect diversity in geographic region, organizational size, and organizational complexity (Table 1). National program officials assured us that the range of MOVE! program activity among participating facilities, as indicated by growth in the number of new program participants and level of program activity (eg, number of program participants receiving individual face-to-face or telephone counseling or group education), reflected the wide range of MOVE! program activity among VHA facilities.

This study was reviewed for human subjects protection and approved by all participating VHA facility institutional review boards and by the review boards of the 2 coordinating centers.

### Data collection

From 2007 through 2010, a researcher (B.J.W.) with 15 years of experience conducting qualitative research, interviewed 68 MOVE! representatives. He asked each VHA facility to identify the MOVE! coordinator, the program's physician champion (formally appointed), the facility manager directly responsible for overseeing the program, an opinion leader in primary care, and 3 or 4 members of the program's multidisciplinary team (Table 2). Of the 74 people contacted, 5 did not respond to recruitment e-mails, and 1 could not be reached because she was on maternity leave. The interviewer had no previous relationship with interview participants. He used semistructured interview guides informed by the study's conceptual framework to gather information about the program's staffing, structure, and operations and facilitators and barriers of program implementation (Appendix). The 30- to 60-minute telephone interviews were recorded with permission from the participants and transcribed verbatim.

### Data analysis

Analysis proceeded in 3 steps. First, we used Atlas.ti version 5.0 qualitative data analysis software (Scientific Software Development GmbH, Berlin, Germany) to code the data. Using a codebook informed by the conceptual framework, 2 investigators independently coded the transcripts, compared their coding, and reconciled coding discrepancies through discussion until consensus was reached. Second, we conducted a within-case analysis of facilitators and barriers for each facility. We generated reports of all text segments for each code and wrote memoranda in which we assessed the degree to which the construct emerged in the data (its "strength"), identified themes in the coded data for the construct, and assessed the degree to which the construct positively or negatively affected implementation (its "valence"). We then created a checklist matrix to visually display the construct valences and support the identification of patterns within medical facilities (16). Finally, we developed a conceptually clustered matrix to enable a between-case analysis of facilitators and barriers by construct (16). Two investigators independently conducted the within- and between-case analyses, compared results, discussed findings, reconciled discrepancies, and produced a final conceptually ordered matrix.

## Results

All 10 VHA medical facilities encountered facilitators and barriers as they implemented MOVE! (Table 3). Although some facilities reported more barriers than others, no facility had barrier-free implementation. Among the 10 facilities, the organization's readiness for change and the presence of an innovation champion most consistently served as facilitators of MOVE! implementation. Other organizational factors, such as resource availability and innovation-values fit, either acted as barriers to implementation or exerted mixed effects (Table 4) on implementation. None of the barriers observed prevented any of the 10 facilities in this study from implementing MOVE! However, interview participants cited the barriers as obstacles to be overcome, worked around, or accepted as limits on the program's scope or scale.

All facilities either had an existing weight-management program or had participated in the pilot phase of MOVE! before issuance of VHA policy. Moreover, all facilities knew that the VHA central office would soon hold them accountable for their obesity screening rates (a key factor leading to increased demand for MOVE! treatment). However, preexisting weight-management programs at 3 facilities provided limited preparation for MOVE! because they focused primarily on healthful eating and offered only group education. In 1 facility, previous programs were perceived as failures, which undermined organizational readiness. Even with pilot-phase experience, 2 facilities struggled to offer the full range of tiered treatment options of MOVE!. Delaying accountability for obesity screening gave facilities time to implement MOVE!; the delay, however, had the unintended effect of reducing the sense of urgency during the interim period, leading to slower MOVE! implementation than interview participants at 2 facilities had desired. Finally, obesity screening rates were added to an already long list of performance indicators at 2 facilities, which may have diluted the motivational effect of such accountability.

Interview participants often, but not always, characterized the facility's senior managers (eg, facility director, chief of staff, facility chief nurse, and chief administrative officer) as supportive of MOVE!. In 2 facilities, senior managers allocated resources for hiring staff or purchasing materials during the pilot phase or immediately after the national launch. However, in 4 other facilities, senior management support did not translate into resource allocation until facilities became accountable for their obesity screening rates. Moreover, the support of service-line chiefs for MOVE! was highly variable, ranging from enthusiasm to passive acceptance to skepticism. (Service-line chiefs are the formal leaders of clinical service lines [eg, primary care service-line chief]; they report to senior managers.) Service-line chief support varied as a function of where the MOVE! program was based administratively. In 2 facilities where MOVE! was based in nutrition service, for example, support from the primary care service-line chief was sometimes tepid. In 3 facilities, interview participants attributed variable service-line chief support as a barrier to creating and sustaining a multidisciplinary team approach to MOVE! program delivery.

In several facilities, interview participants cited limited resource availability as a significant barrier to MOVE! implementation. Three facilities praised the toolkit that NCP developed for MOVE! implementation and delivery. The national program launch, however, provided no additional funding for facilities to implement MOVE!. With no additional funding, 5 facilities launched MOVE! by assigning existing clinical staff the additional duty to implement and deliver MOVE!. When facilities became accountable for their obesity screening rates, facility managers at 2 facilities proved more receptive to requests to hire full-time staff for MOVE!. In all 10 facilities, however, MOVE! relied heavily on the staff who were personally committed to supporting and delivering the program in addition to performing their other clinical or administrative duties. Four facilities coped with limited staffing resources by involving psychiatric residents, psychology interns, and nutrition students from nearby universities. Interview participants generally reported that MOVE! is understaffed in their facility and that the understaffing limits the number of veterans served, the range of tiered treatment options, and the multidisciplinary approach. In 5 facilities, for example, interview participants reported little or no staff support in physical activity disciplines (eg, recreational therapy, physical therapy, occupational therapy). Five others reported shortages in behavioral health disciplines (eg, psychology, social work).

VHA policy required all facilities to assign a physician champion for MOVE! In most facilities, interview participants reported that the physician champion was actively engaged in MOVE! and served as a respected ambassador for the program among primary care physicians and an influential advocate for additional resources. In 2 facilities, however, the physician champion was described as uninvolved in MOVE! or passive as a spokesperson for the program. In these facilities, interview participants sometimes identified the MOVE! coordinator or another MOVE! staff member as an innovation champion. These people, however, did not have the position, prestige, or influence of the physician champion.

Primary care physicians are expected to screen patients for obesity, counsel them about the health risks and consequences of obesity, and refer them to MOVE! if they seem interested or ready. Interview participants at 7 facilities noted that primary care physicians strongly believe in the value of prevention and perceive weight management as necessary for reducing illness among their patients and to VHA as a health care system. As a comprehensive, multidisciplinary weight-management program that offers tiered treatment options tailored to patient needs and interests, the MOVE! program fits the values of many primary care physicians. However, interview participants at 4 facilities noted that some primary care physicians doubt the program's efficacy to produce and sustain enough weight loss to make a noticeable impact on patients' health. This skepticism, plus the urgency of patients' more pressing medical issues, led to less support from some physicians.

All 10 facilities attempted to tailor MOVE! to better fit their organization's capacity to implement it. These modifications included adding or removing clinical reminders for obesity screening, tailoring procedures for enrolling patients, and offering various levels of the MOVE! program at a facility. Eight facilities noted that primary care nurses and physicians felt that tasks associated with MOVE!, such as the clinical reminder to screen for obesity or attending multidisciplinary meetings, were time consuming and burdensome to already heavy workloads. Two facilities decided to remove the clinical reminder altogether.

Enrolling patients in MOVE! was challenging for some facilities. One facility reported patient reluctance to participate in a weight-loss program. Additionally, 4 facilities had difficulty motivating patients to practice behavior changes, such as exercising and eating healthfully, outside of the MOVE! classroom. Implementation of the most basic treatment option — self-management supported by frequent telephone contact — varied among facilities. Four facilities discontinued this level because they had difficulty reaching people by telephone and it was time consuming for staff and volunteers to make calls. One facility could make initial telephone calls but noted that staff availability limited the number of follow-up calls. Another found this level was more convenient for patients living farther away.

## Discussion

Organizational facilitators and barriers played a salient role in the implementation of MOVE! — the only nationally implemented, evidence-based weight-management program that focuses on reducing obesity rates among US veterans receiving care at VHA facilities. Of the 6 organizational factors examined in this study, organizational readiness for change and innovation champions were the most consistent facilitators of MOVE! implementation. Management support, resource availability, innovation-values fit, and innovation-task fit either acted as barriers to implementation or exerted mixed effects on implementation.

Our findings contribute to a limited body of research on the organizational context of innovation implementation in health care settings (17,18). A study with similar findings (19) observed that resource limitations posed a substantial barrier to the implementation of quality improvement and patient safety interventions in infection prevention. Our results suggest that organizational accountability through explicit performance measurement can prompt health care organization leaders to allocate scarce resources to support program implementation and spur program staff to find creative solutions to resource constraints. Several studies indicate that informal, emergent innovation champions play a role in innovation implementation (9,20-24). Our results suggest that formally appointed innovation champions can also aid implementation by helping secure resources, overcome obstacles, and encourage innovation.

This study had several limitations. Case study research emphasizes depth over breadth and insight over generality (12,15). Ten cases do not provide a strong basis for statistically generalizing study results to all VHA facilities. Although national program officials (L.C.K. and L.S.K.) report many VHA facilities encountered the same or similar organizational facilitators and barriers as those identified in this study, a national survey of randomly sampled VHA facilities would be needed to document the frequency and distribution of facilitators and barriers. As is true of all research, case study research involves an irreducible element of expert judgment. We used time-honored case study research methods, but we cannot discount the possibility that investigator bias in interpretation influenced our results.

We suggest 2 directions for future research. First, the theory and practice of the multilayered complexities of management support need to be understood. Senior management support is often cited as necessary for innovation implementation (14,25-29), but our study shows that support from middle managers (eg, service-line chiefs) and even direct supervisors can also aid or hinder implementation. Second, innovation champions are often conceptualized as people who, driven by passion and enthusiasm, not formal designation, step outside of their organizationally prescribed roles to advocate for innovations (9,20-24). Our study shows, however, that formally designated innovation champions promoted implementation in many facilities; informal champions surfaced only when formally designated champions left a gap to be filled. The emergence of informal champions, rather than being lauded, should perhaps be considered a sign that the organization's formal roles, structures, and policies are not aligned with its goals for program implementation. This conjecture could be empirically investigated.

We also learned 2 practical lessons that may help other health care or public health systems to implement new programs amid competing organizational priorities and a lack of new resources. First, organizational leaders directing implementation of new programs must put into place powerful, mutually reinforcing policies and practices that make implementation expected, supported, and rewarded. Such policies and practices include setting measurable goals for implementation, instituting a realistic schedule for meeting those goals, monitoring progress against goals, recognizing those who meet goals, and holding accountable those who do not. These policies and practices must be clearly and consistently communicated, and they must command the attention of those charged with implementation. Second, the policies and practices must cascade throughout the multiple levels of organizational hierarchy to form an aligned, interlocking implementation strategy. Otherwise, an implementation gap arises between top management and the front line of service provision to veterans.

## Figures and Tables

**Table 1.** Veterans Health Administration (VHA) Medical Centers Included in Qualitative Study on Implementation of the MOVE! Weight-Management Program, United States, 2007-2010

**Medical Facility**	Census Region	No. of Unique Outpatient Visits^a^	**Facility Complexity Rating** b	**No. of New Unique MOVE! Patients** c	**No. of Unique MOVE! Visits** c ^,^ d
1	West North Central	37,221	1C	207	2,977
2	West South Central	85,112	1A	81	409
3	East North Central	41,479	1B	195	758
4	New England	63,294	1A	104	581
5	East North Central	54,494	1A	427	2,914
6	West	65,771	1A	374	1,074
7	New England	54,401	1A	129	960
8	Mountain	39,869	1B	259	574
9	West	63,514	1A	224	358
10	East North Central	77,968	1A	632	1,706

a Data were obtained for fiscal year 2006 from the VHA Service Support Center Unique Patient Data Cube (unpublished data).

b The VHA categorizes VHA Medical Centers according to a defined complexity model for the purposes of performing program and organization analyses, making decisions on organizational structure, and setting senior executive pay levels. The model uses data on patient population served (including numbers served and patient risk as measured by the diagnostic cost group), clinical services complexity (eg, intensive care units, specialized clinical programs), and the scope of the graduate medical education and research enterprise of the facility. Facilities are categorized into 1 of 5 complexity levels: IA (most complex), IB, IC, 2, or 3 (least complex).

c Data were obtained for fiscal year 2006 from the VHA Service Support Center MOVE! Visits Data Cube (unpublished data).

d Visits include group, individual, and telephone communication. Visits are identified through the use of a unique administrative code required by VHA policy.

**Table 2:** Number of Interview Participants, by Veterans Health Administration (VHA) Facility and Organizational Role, Qualitative Study on Implementation of the MOVE! Weight-Management Program, United States, 2007-2010

Facility	**Organizational Role** a					

	Coordinator	Physician Champion	Facility Manager	Multidisciplinary Team Member	Opinion Leader	Total
**1**	2	1	1	3	1	8
**2**	1	0^b^	1	3	1	6
**3**	1	1	0^c^	3	0^c^	5
**4**	1	1	1	3	1	7
**5**	1	1	1	3	1	7
**6**	1	1	0^c^	5	1	8
**7**	1	1	1	3	1	7
**8**	1	1	1	3	1	7
**9**	1	1	1	1^d^	1	5
**10**	1	1	1	4	1	8
**Total**	11	9	8	31	9	68

a The coordinator is the clinical staff person responsible for program coordination, communication, and reporting. The physician champion is responsible for facilitating program implementation and overseeing the clinical aspects. The facility manager is the administrator directly responsible for overseeing the program; facility managers had different titles in different VHA facilities (eg, associate chief of staff for ambulatory care, primary care service line manager, nutrition/food service chief). Multidisciplinary team members are clinical staff from the 4 core disciplines involved in program delivery: dietetics, primary care, physical activity, and behavioral health. The opinion leader is a primary care physician who is not directly involved in the program but is considered influential in primary care.

b Physician was on maternity leave; we were unable to reach her.

c Participant did not respond to recruitment e-mail.

d Two interview participants did not respond to recruitment e-mail.

**Table 3:** Organizational Factors Associated With Implementation of MOVE! Weight-Management Program, United States, 2007-2010

	Facility									

	1	2	3	4	5	6	7	8	9	10
Organizational readiness^a^	+	+	+/−	+/−	+	+	+	+	−	+
Management support^b^	+/−	−	−	+/−	+	+/−	+/−	+	+/−	+/−
Resource availability^c^	+/−	−	+/−	+/−	+/−	+/−	+/−	+	−	+/−
Innovation champion^d^	+	−	+	+	+	+	+	+/−	+/−	+
Innovation-values fit^e^	+/−	+/−	−	−	+/−	+/−	+	+/−	−	+/−
Innovation-task fit^f^	+/−	−	−	−	+/−	+	+	+/−	−	+/−

Abbreviations: + indicates factor was present and favorable for implementation; −, factor was absent or unfavorable for implementation; +/−, factor was present but mixed (favorable and unfavorable) for implementation.

a Refers to the extent to which expected implementers and users of an innovation are psychologically and behaviorally prepared to make the necessary changes in organizational policies and practices.

b Refers to managers' shared resolve to pursue courses of action that promote the successful implementation of the innovation.

c Refers to the accessibility of financial, material, or human assets that can be used to support initial and ongoing innovation use.

d Refers to a charismatic person who supports the innovation, thus overcoming the indifference or resistance that a new idea often provokes in an organization.

e Refers to the extent to which targeted employees perceive that innovation use will fulfill their values.

f Refers to the extent to which the innovation is compatible with task demands, work processes, and organizational capabilities.

**Table 4:** Facilitators and Barriers to Implementing MOVE! in Veterans Health Administration (VHA) Medical Facilities, United States, 2007-2010

Construct	Facilitator	Barrier
**Organizational readiness**	Prior weight-management programs and MOVE! pilot prepared sites for MOVE!	Prior programs provided only partial preparation (eg, nutrition focus, classes only)
	Impending performance indicator created motivational context for implementation	Impending performance indicator part of much larger set of performance indicators
**Management support**	Managers and chiefs generally supportive	Service-line chief support highly variable
	Managers (re)allocate limited resources	Senior managers generally unfamiliar with MOVE!
**Resource availability**	VHA's National Center for Health Promotion and Disease Prevention generated useful program materials and implementation tools	Program underresourced in clinical and administrative staffing
	Committed staff and clinical trainees filling staffing gap	Space for MOVE! often too small, poorly configured
**Innovation champion**	Physician champion is credible ambassador with physician and management audiences	Physician champion engagement in MOVE! highly variable across facilities
	Physician champion sometimes a powerful advocate for resources	Physician champion sometimes lacks political savvy and bargaining skills
**Innovation-values fit**	Prevention is a moderate- to high-intensity value in VHA	Physicians somewhat skeptical about program's efficacy
	Weight management viewed as important to improving health	Prevention competes with acute care for attention and resources
**Innovation-task fit**	Multiple program levels fit veterans' needs	Veterans' motivational readiness highly variable
	Clinical reminder provides timely cue to action	Primary care workload is overwhelming

## Appendix. Interview Guide for Qualitative Study on Implementation of the MOVE! Weight-management Program, Veterans Health Administration, United States, 2007-2010

**Organizational readiness for change** refers to the extent to which targeted organizational members (especially the implementers and intended users) are psychologically and behaviorally prepared to make the changes in organizational policies and practices that are necessary to put the innovation into practice and to support innovation use.

**Table:**

	Facility Manager	MOVE! Coordinator	MOVE! Physician Champion	MOVE! Multidisciplinary Team	Opinion Leader
What prompted your facility to adopt MOVE!? Was the decision externally driven or internally motivated? What issues did you all consider in deciding to adopt MOVE!? What were the "pros" and "cons," so to speak?	X	X	X		

How committed were your facility's senior managers? How committed were your facility's service line chiefs? How committed were your facility's [providers, clinicians]? Where there any important groups or individuals who seemed unsure or perhaps reluctant?	X	X	X	X	X

Prior to MOVE!, what kinds of services did your facility offer to patients who were overweight or obese? Were these services multidisciplinary? Did people see MOVE! as a better alternative? Why or why not?	X	X	X	X	X

How confident were you that your facility could implement MOVE! successfully? What did "successful implementation" mean for you? Were you more confident about some elements of MOVE! than others? What prompted you to feel this confident? Who shared your level of confidence? Who did not?	X	X	X	X	X

**Management support** refers to facility or VISN managers' shared resolve to pursue courses of action that promote the successful implementation of the innovation. Although titles vary, management includes facility director, facility chief of staff, facility chief nurse, facility chief administrative officer, facility service line chiefs, VISN network director, VISN chief medical officer, and VISN clinical leads. Although some MOVE! coordinators wear "management hats," the coordinator role is not considered a management position.

**Table:**

	Facility Manager	MOVE! Coordinator	MOVE! Physician Champion	MOVE! Multidisciplinary Team	Opinion Leader
How supportive of MOVE! are your facility's senior managers? Can you think of specific things that they have done or said that demonstrate support, or lack of support, for MOVE!? Are some more supportive than others? How has their level of support changed since you first got started? What accounts for these changes?	X	X	X	X	X

How supportive of MOVE! are your facility's service line chiefs? Can you think of specific things that they have done or said that demonstrate support, or lack of support, for MOVE!? Are some more supportive than others? How has their level of support changed since you first got started? What accounts for these changes?	X	X			

**Resource availability** refers to the accessibility of financial, material, or human assets that can be used to support initial and ongoing innovation use.

**Table:**

	Facility Manager	MOVE! Coordinator	MOVE! Physician Champion	MOVE! Multidisciplinary Team	Opinion Leader
Are there enough providers in the core disciplines in your facility to provide MOVE! in your facility? Are there enough clinicians to increase the current level of MOVE! in your facility? If not, which clinical disciplines are in short supply? What accounts for that? What could be done to improve provider availability?		X	X	X	X

How satisfied are you with the *space* available for group meetings? Has the quality or quantity of space affected the number, frequency, or size of group sessions? What needs for space exist? What could be done to address these needs for space?		X		X	

How satisfied are you with the *equipment* available to support MOVE! (eg, computers, printers, and furniture)? Has the quality or quantity of equipment affected MOVE! implementation? What needs equipment exist? What could be done to address these needs for equipment?		X		X	

Does your VISN provide financial resources for MOVE! beyond usual patient care dollars? If so, how much and for what purpose? If not, has your facility requested it? What happened? Likely to change?	X	X			

**Implementation policies and practices** refer to the plans, practices, structures, and strategies that an organization employs to put the innovation into place to support innovation use.

**Table:**

	Facility Manager	MOVE! Coordinator	MOVE! Physician Champion	MOVE! Multidisciplinary Team	Opinion Leader
Please describe how you have implemented MOVE!. How are patients screened for BMI? Who determines eligibility? Gives risk education? Offers MOVE!? How do patients fill out MOVE!23? Who reviews MOVE!23 results with patients? Who helps patients set goals? Who schedules follow-up MOVE! appointments? Who does the follow-up? How is it done: primary care, consults, groups? Who tracks patients' progress?		X	X (first 2 bullets only)	X	X (first 2 bullets only)

Does your facility do "same day" enrollment? If so, what does it take to make that work? How well is it working? If not, have you considered it? What would it take to do it?		X	X	X	

How do providers involved in MOVE! communicate and coordinate with each other? [methods, frequency, quality of communication]		X	X	X	

Have you established clinic profiles for MOVE!-related appointments? Do you have a clinical reminder to assist with screening? Do you have the toolbar launch for the MOVE!23 installed on CPRS? Do you have a MOVE!-related progress note title in the list of titles? Can you query your local VISTA for all patients enrolled in MOVE! for tracking purposes?		X	X	X	

How does your facility train new providers in MOVE!?		X	X	X	

What ongoing education and training does your facility provide with regard to MOVE!? Obesity and overweight?		X	X	X	

Has your facility marketed MOVE! to patients? If so, what have you done? What works? What doesn't? If not, do you plan to do so? What would it take to do so?		X	X	X	

How often do providers receive feedback on facility-level performance on MOVE!? What kinds of feedback do they receive? How do they get that feedback?	X	X	X	X	

How much time or effort is required to provide MOVE! on a daily basis? Did getting MOVE! implemented take more time or effort than expected? Has the amount of time or effort to provide MOVE! decreased as your facility has gained more experience with MOVE!?		X	X	X	

**Innovation-task fit** refers to the extent to which the innovation is compatible with task demands, work processes, and organizational capabilities.

**Table:**

	Facility Manager	MOVE! Coordinator	MOVE! Physician Champion	MOVE! Multidisciplinary Team	Opinion Leader
What aspects of MOVE! are most feasible? What makes them so?		X		X	

What aspects of MOVE! are least feasible? What makes them so?		X		X	

How could MOVE! be redesigned to make it more feasible?		X		X	

**Implementation climate** refers to organizational members' shared perceptions of implementation policies and practices in terms of their meaning and significance for innovation use.

**Table:**

	Facility Manager	MOVE! Coordinator	MOVE! Physician Champion	MOVE! Multidisciplinary Team	Opinion Leader
How involved is the physician champion? What does he or she do? How visible is he or she? Could he or she make things happen to support MOVE!? Does he or she make things happen?	X	X		X	X

How involved is the facility MOVE! coordinator? What does he or she do? How visible is he or she? Could he or she make things happen to support MOVE!? Does he or she make things happen?	X		X	X	X

Do clinicians here feel that they are expected to participate in MOVE!? Do they know what they are supposed to do? Do they feel that they have the support they need? Do they feel that their participation in MOVE! is recognized and valued?	X	X	X	X	X

Do providers here feel that they are expected to participate in MOVE!? Do they know what they are supposed to do? Do they feel that they have the support they need? Do they feel that their participation in MOVE! is recognized and valued?	X	X	X	X	

**Innovation-values fit** refers to the extent to which targeted employees perceive that innovation use will foster the fulfillment of their values. Values are concepts or beliefs that a) pertain to desirable end-states or behaviors, b) transcend specific situations, and c) guide the selection and evaluation of behavior and events.

**Table:**

	Facility Manager	MOVE! Coordinator	MOVE! Physician Champion	MOVE! Multidisciplinary Team	Opinion Leader
What motivates provider to participate in MOVE!? Do providers feel comfortable with MOVE!? Why or why not? What do they like about MOVE!? What do not like?	X	X	X	X	X

In what ways does MOVE! fit with management's priorities? In what ways does MOVE! not fit with management's priorities?	X	X	X	X	X

**Innovation champion** refers to a charismatic individual who throws his/her weight behind the innovation, thus, overcoming the indifference or resistance that a new idea often provokes in an organization.

**Table:**

	Facility Manager	MOVE! Coordinator	MOVE! Physician Champion	MOVE! Multidisciplinary Team	Opinion Leader
Is there a particular provider, clinician, or manager who really goes above and beyond the call of duty to make MOVE! succeed? Is there someone who does far more than what he or she is expected to do?	X	X	X	X	X
